# Bladder cancer discussed on the internet: a systematic analysis of gender differences of initial posters on an online discussion board

**DOI:** 10.1186/2193-1801-2-445

**Published:** 2013-09-08

**Authors:** Yannick Lippka, Oliver Patschan, Tilmann Todenhöfer, Christian Schwentner, Andreas Gutzeit, Axel S Merseburger, Marcus Horstmann

**Affiliations:** Medical School, Otto-von-Guericke University Magdeburg, Leipziger Str. 44, Magdeburg, 39120 Germany; Department of Urology, Skåne University Hospital, Lund University, Jan Waldenströms gata 5, Malmö, 20502 Sweden; Department of Urology, Eberhard Karls University Tuebingen, Hoppe - Seyler– Str. 3, Tuebingen, 72076 Germany; Department of Urology and Urologic Oncology, Hannover Medical School, Carl-Neuberg-Str. 1, Hannover, 30625 Germany; Department of Urology, Jena University Hospital, Lessingstr. 1, Jena, 07743 Germany; Institute of Radiology, Kantonsspital Winterthur, Brauerstr. 15, Winterthur, 8401 Switzerland; Institute of Radiology, University Hospital of Salzburg, Müllner Hauptstrasse 48, 5020 Salzburg, Austria

## Abstract

**Objectives:**

To evaluate gender differences of initial posters in threads dealing with bladder cancer on an online discussion board.

**Method:**

529 threads opened between 09/2005 and 03/2012 were screened on the largest German speaking bladder cancer online discussion board. 366 threads fulfilled the requirements for this study. Gender, age, number, status of concern and oncological situation of initiating posters as well as their motives and language style were analyzed following a standardized protocol.

**Results:**

Threads were initiated in 45% (164/366) by men and in 55% (202/366) by women. Mean age of male initiating posters was 50 years and of female posters 44 years (p < 0.001). Of males 80% (132/164) were concerned patients and 20% (32/164) relatives or friends. Of females they were 39% (78/202) and 61% (124/202), respectively (p < 0.001). In general motives for initial posting were focused on medical information and did not differ between both genders. 81% of the posters asked for medical information or therapeutic recommendations regarding diagnosis, treatment and prognosis. However, women significantly more often expressed their wish for emotional support (p = 0.034) and in tendency wanted to share their experiences with others (p = 0.057). Language analysis revealed that women significantly more often used a tentative language style than men (p = 0.003).

**Conclusion:**

Even though women are less often affected by bladder cancer, they are more active –especially for their concerned family members - on the evaluated discussion board than men. Whereas both genders equally often ask for medical information, women more often want to share their experiences and look for emotional support.

## Introduction/objective

Bladder cancer (BC) remains a major health problem around the globe (Ploeg et al. 2009). In Germany about 28.000 patients are newly detected with bladder cancer and about 6000 die of it each year (Robert Koch-Institut Hrsg 2012). Due to exposure differences and other not yet completely understood gender differences men are affected 3-4 times more often than women (Horstmann et al. 2008). At first detection bladder cancer usually requires transurethral resection of the bladder (TURB). Follow up of non-muscle invasive bladder cancer (NMIBC) then consists of repeated cystoscopies and in case of recurrence TURBs. In muscle invasive disease (MIBC) or highly aggressive NMIBC, radical cystectomy with urinary diversion is the standard therapy (Babjuk et al. 2011; Stenzl et al. 2011). In both situations invasive and often repeated treatment modalities and the necessity of a constant follow-up make bladder cancer a disease with high costs (Sievert et al. 2009) and a tremendous impact on quality of life representing a huge psychological burden (van der Aa et al. 2008). In order to cope with this, patients might seek professional support, but also have the possibility to establish contact with peers. In Germany this is offered by local support groups with face-to-face contact. However, in recent years the Internet has become an additional place for opinion sharing with easy access to disease specific online discussion boards (Huber et al. 2011; Owen et al. 2007). In several studies such a peer-to-peer communication has been shown to empower patients (van Uden-Kraan et al. 2008; van Uden-Kraan et al. 2009), but still until to date its use remains relatively limited (Van Uden-Kraan et al. 2011). Nevertheless especially online discussion boards have become a subject of research in recent years, because they offer a unique access to an otherwise hidden patient-to patient communication (Eysenbach et al. 2004). In urology online discussion boards have only been analyzed in prostate cancer in comparison to online discussion boards of breast and ovarian cancer until now. This repeatedly revealed differences of men and women discussing their disease with in general men being more focused on medical information and women on emotional support (Gooden & Winefield 2007; Sullivan 2003; Mo et al. 2009). Because in contrast prostate cancer bladder cancer affects both genders and gender is an important topic in the epidemiology and biology of bladder cancer (Fajkovic et al. 2011; Gakis & Stenzl 2013; May et al. 2012; Kluth et al. 2012), we evaluated if gender differences were also observable on a mixed gender discussion board for bladder cancer. We considered such an analysis worthwhile as it might help clinicians to better understand the needs, thoughts and feelings of their patients and for bladder cancer online discussion boards have not been evaluated yet.

## Materials and methods

For the purpose of gender evaluation only one large national online discussion maintained by German support groups: http://www.forum-blasenkrebs.net was considered suitable and therefore chosen for evaluation. At the time of data collection it included 3043 threads with 44709 postings and 1542 registered users. Inclusion criteria for the present evaluation were threads of posters dealing with bladder cancer either started by patients themselves or members posting for concerned patients. This allowed evaluating threads in a standardized situation and limited the evaluations to a reasonable extend. Threads posted between 09/2005 and 03/2012 were evaluated in the subdomain of the general bladder cancer forum, the non-muscle invasive and the muscle invasive forum. Exclusion criteria were threads started by members of the online support group (proxy) or off-topic threads, i.e. threads that were initiated by users who had already started a thread. Both open and closed threads were included. A thread was considered as closed if it had not received postings during the last 30 days. In the eligible threads the characteristics and status of initial posters were evaluated. This included: gender, age and status of concern regarding bladder cancer of initial posters and - if not the same person - the patient being discussed. If possible, it was differentiated between patients with NMIBC and MIBC. Motives and content of initial postings were evaluated differentiating between medical questions regarding diagnosis, treatment and prognosis, the wish for treatment advice and the wish to share experiences. Language and key words of initial postings were evaluated separately.

Analyses were done following a standardized protocol established by YL and MH according to the principals of the grounded theory (Chen & Boore 2009). Herein a numbered coding system was used for different qualities of different evaluated aspects. This coding system allowed that different qualities of one aspect were attributed to one posting at the same time, meaning that for example a posting might contain both a medical question and at the same time the wish for emotional support. All threads were evaluated in consensus by both referees in a joint session. Results were then systematically recorded in a database. For statistical comparisons of continuous variables the Student’s *t*-test for independent samples was applied and for comparison of categorical values a binomial or a two-sided Fishers exact test. A p-value of < 0.05 was considered to show a significant difference (JMP version 10.0; SAS, Cary, NC, USA).

The ethical board of the Medical School of Hannover approved the study and responsible administrators of the online support group were informed about the project. The fact that posters were not informed about the analysis was not considered as a problem, because all data were analyzed anonymously and were publically available.

## Results

### Eligible threads

All in all 529 threads were screened of which 69% (366/529) were eligible for the present study. 3.5% (18/529) threads were excluded as being written by proxy and 27.5% (145/529) as being off-topic. Of the 366 eligible threads 59% (216/366) were dealing with NMIBC, 31% (113/366) with MIBC. In 10% (37/366) the stage of the disease was not known. Mean length of initial postings was 194 ± 148 words and not different between male and female posters (m: 192, f: 196). Initial posting resulted in conversations of in mean 13 postings per thread with no gender differences (m: 14, f: 13). Threads were viewed in mean 1746 ± 1573 times either by posters or by lurkers (van Uden-Kraan et al. 2008), i.e. persons that follow the discussions without actively taking part in it. Of these viewers the gender and the amount of lurkers was not evaluable.

### Gender and status of initial posters

Of the evaluated threads 45% (164/366) were initially posted by men and 55% (202/366) by women (p = 0.053). Of men, 80% (132/164) were concerned patients and 20% (32/164) relatives or friends. Of women they were 39% (78/204) and 61% (124/202), respectively (p < 0.0001). In the group of relatives or friends posting for others (n = 156) the number of females was significantly higher than that of males (w: n = 123, 80% vs. m: n = 33, 20%, p < 0.0001). In this group daughters or daughters in law were most often posting for their relatives (56% (87/156)). Evaluated mean age of men as initial posters was 50 (range 19 – 75) years and of women 44 (23-77 range) years (p < 0.01). The mean age of the patients being discussed by others was for men 65 (range 39 – 90) years and for women 66 (range 44 - 90) years (Table 1).

**Table 1:** **Data of initial posters differentiated between men and women**

	Men	Women	p-Value
**Characteristics of initial posters:**			
Initial postings opening threads, n (%)	164 (45)	202 (55)	0.053^#^
Mean age of initial posters, y (range)	50 (19-75)	44 (23 - 72)	<0.0001*
Threads opened by concerned patients, n (%)	132 (80)	78 (39)	<0.0001*
Threads opened for concerned patients n, (%)	32 (20)	124 (61)	
**Content:**			
Asking for medical information, n (%)	139 (85)	158 (78)	0.139†
Wish for emotional support, n (%)	63 (38)	101 (50)	**0.034†**
Wish to share experiences, n (%)	68 (41)	104 (51)	0.057†
**Language:**			
Questions, n (%)	126 (77)	162 (80)	0.367†
Statements implying questions, n (%)	21 (13)	33 (16)	0.376**†**
Pure statements, n (%)	17 (10)	7 (4)	**0.010†**
Tentative language, n (%)	68 (41)	116 (57)	**0.003†**
Use of acronyms	Rarely	Rarely	
Use of medical terms	Often	Often	
**Responses and feedback:**			
Mean number of postings per thread (n)	14	13	0.410*
Mean views per thread (n)	1836	1675	0.934*

Data of initial posters on an online bladder cancer discussion board differentiated between men and women, ^#^ - binomial test,*- Student’s *t*-test, **†**- two sided Fishers exact test.

Significant differences are marked in bold numbers.

### Motives for initial postings

At the time of initial posting most patients had just previously a TURB (75% (273/366)). Of them the majority had had their first TURB (83% (227/273)). The others had had re-interventions. Motives for initial posting were in 81% medical questions including in 43% questions about the histopathological findings, in 37% questions about treament, in 43% questions about diagnostics and prognosis and in 31% the wish for further treatment advice. Other motives often overlapping with medical questions were in 47% the wish to share experiences and in 45% to receive emotional support. Whereas gender analysis of medical questions revealed no significant or in tendency significant differences between men and women, women significantly more often expressed their wish for emotional support (w: 50% (101/202) vs. m: 38% (63/164), p = 0.034) and in tendency significantly more often wanted to share experiences (w: 51% (104/202) vs. m: (41% (68/164), p = 0.057) (Table 1).

### Responses and feedback

492 different persons gave 4596 responses to the evaluated 366 initial postings. Men gave 2443 and women 2647 answers. Mean age of responding posters was not evaluable because it was rarely mentioned on the discussion board. The male-to-female ratio of responders was evaluated according to names chosen by the posters and was 0.8:1 (m: n = 218 vs. f: n = 274). Most responses (31.5% (1604/4596)) were given by the five most active posters (1%, (5/492)), of them 3 were men and 2 women. With regard to addressed topics in initial postings no differences of the mean length of responding threads was observed, also not in the comparison of threads initiated by men and those by women (m: 14 threads vs. w: 13 threads, p = 0.41) (Table 1).

### Language

From point of view of language the majority of postings included direct questions (78% (285/366)). This did not differ between both genders. However men significantly more often gave pure statements without including questions in their initial postings than women (p = 0.0103). 50% of the initial postings (184/366) were written in a tentative language that was defined by the subjunctive form or words expressing uncertainty, like: maybe, eventually and so on. In comparison significantly more women used this tentative language than men (w: 57% vs. m: 41%, p = 0.003). In contrast to other online discussion boards key word analysis did not reveal a regular use of acronyms instead of medical terms by both genders. The words “tumor” or “cancer” as well as other medical terms were regularly used without differences between both genders (Table 1).

## Discussion

Today, disease specific online discussion boards are an important (Neuhauser & Kreps 2008) but still underestimated source of information and emotional support for patients and their relatives or friends. Because they offer a unique possibility for analyzing a patient-to-patient or peer-to-peer communication they have also become the subject of scientific interest (Eysenbach et al. 2004). In the present study the most important and most highly visited German speaking bladder cancer discussion board: http://www.forum-blasenkrebs was evaluated with focus on gender differences of initial posters. The rationale for this study was to verify if differences between the online activity and communication of men and women as described in other cancer types (Gooden & Winefield 2007; Gray et al. 1996; Kiss & Meryn 2001) also exist in bladder cancer. Such a profound analysis might help clinicians to better counsel their patients.

Generally the online board was well accepted, as shown by the high number of visits and threads and was almost equally used by both genders. In comparison to a general bladder cancer population, however, with a male-to-female ratio of 4-3:1, it revealed a higher online activity of women than of men. This was to our knowledge not yet shown in the literature in a cancer that affects both genders to a different extend. In the present evaluation the higher online activity of women was mostly due to the significantly higher number of healthy females posting for their male partners or family members than vice versa. Especially younger females (daughters or daughters in law) very often posted for their fathers or fathers in law whereas men rarely posted for their female partners. Similar observations were already made by Gray et al. and Kiss et al. who found women to be more active in caring for their male family members in self-help groups of prostate cancer patients than men in case of their partners breast cancer (Kiss & Meryn 2001; Gray et al. 1997).

Regarding age we found the mean age of concerned initial posters (w: 47 y, m: 52 y) to be about 15 – 20 years below a general bladder cancer population and about the same as in other discussion boards (van Uden-Kraan et al. 2009). In case of bladder cancer the present results showed that mainly younger bladder cancer patients or their family members used the discussion board whereas old or very old patients remained underrepresented. Remarkably, however, the majority of posters were persons, who did not grow up with the Internet, demonstrating its high acceptance as a place for disease specific communication also in these age groups. Interestingly the mean age of patients, discussed about by relatives or friends, exactly matched with the mean age of patients with newly detected bladder cancer (66 y). This together with the fact that most cancers were newly detected bladder cancers led us to the conclusion, that especially patients and family members directly after a primary transurethral resection have a high need of information and counseling.

From point of view of addressed topics and motives for initial posting we found no differences between men and women and observed that most posters mainly looked for medical information. However women more often included their wish for emotional support and to share experiences in their postings than men. Regarding this (Mo et al. 2009) recently described in their review of 12 studies diverging findings with some studies showing gender differences and others not. Even though they summarized that gender differences regarding emotional aspects were probably more pronounced in comparison of single gender discussion boards, our study also revealed significant gender differences on a mixed gender discussion board.

Evaluating language most initial postings included open or direct questions asking for medical information. Only a few postings were pure statements. Men however used this category significantly more often than women. Similar to Huber et al. many initial posters (50%) used a tentative language on the present online board (Huber et al. 2011). We mainly interpreted this as an expression of their uncertainty in their role as novices on the discussion board. Additionally, we observed that women significantly more often used a tentative language than men, which underlined principal differences in the communication of both genders. Whereas in other studies key word analysis revealed a frequent use of acronyms by posters instead of medical terms (Huber et al. 2011; Seale et al. 2006), this was not the case in the present evaluation. Herein irrespective of gender medical terms were often and acronyms rarely used. These findings again reconfirmed our impression that the present discussion board was mainly focused on medical information and less on emotional support.

Answers were given almost equally distributed between both genders with a male to female ratio of 0.8:1 confirming the previously mentioned higher online activity of women than men. Most importantly, however, we found that irrespective of gender, 31.5% of the answers were given by only 1.0% of the most active posters on the present board. This revealed a rather oligarchic than democratic structure of the discussion board and is a critical point especially when it comes to the distribution of medical information and opinions. Similar structures of other online discussion boards were already remarked and criticized by others (Huber et al. 2011). On the present board, however, we had the impression that answers were given with great care and were mostly medically correct.

As the most important limitation of the present study we have to mention the limited generalizability of the present data. Only a limited number of threads, e.g. of initial posters, were analyzed of only one German speaking bladder cancer online discussion board. Additionally only two referees (YL and MH) evaluated all threads, which makes from a methodological point view a personal judgment bias possible. Therefore the present data have to be interpreted with great care.

However, in conclusion we consider these data worth reporting as they analyze for the first time gender aspects in the peer-to-peer communication about bladder cancer on an online discussion board. This revealed that the online discussion board was well accepted and mainly used for medical information by both genders. We thereby had the impression that the online discussion board offers an easy, time- and place independent opportunity to establish rapid peer-to-peer communication for medical information and emotional support. Comparing communication between both genders however we found women to be more active as posters, more likely to seek emotional support and more often to want to share experiences than men. In contrast to that male communication was more focused on information. According to our believes these differences are worth realizing as they imply that - besides known biologic differences- also communicational gender differences exist and have to be taken into account for the treatment of bladder cancer patients.
